# Editorial: “Innovation informs best practices in autism intervention across the lifespan”

**DOI:** 10.3389/fresc.2022.968309

**Published:** 2022-07-29

**Authors:** Gloria K. Lee, Ana Dueñas, Eun-Jeong Lee, Courtenay A. Barrett, Chetwyn C. H. Chan

**Affiliations:** ^1^Department of Counseling, Educational Psychology and Special Education, Michigan State University, East Lansing, MI, United States; ^2^Department of Education and Human Services, Lehigh University, Bethlehem, PA, United States; ^3^Department of Psychology, Illinois Institute of Technology, Chicago, IL, United States; ^4^Department of Psychology, The Education University of Hong Kong, Hong Kong, Hong Kong SAR, China

**Keywords:** lifespan, biopsychosocial, autism, best practices, intervention

Autism spectrum disorder (ASD) is a condition characterized by the core symptoms of limited social communication, restricted interests, and repetitive behaviors (1) and is diagnosed in 1 out of every 44 children (2). ASD is pervasive in nature, thus at different developmental milestones, core symptoms affect varying functional life domains, such as physical and mental health, independent living, education, work and relationships (3–6). Autism is a “spectrum” condition because of its variation in severity, types, and comorbidities that affect each individual differently. The social nature of humanity makes social communicative deficits more adverse among those impacted by ASD. ASD occurs across all ethnic, racial, and socioeconomic groups. Due to the complexity of the condition with many individual and environmental factors, effective intervention is challenging.

Evidence-based practices must address “what intervention works for whom under which conditions” to facilitate a person's positive treatment outcomes (7). Understanding how different attributes influence treatment can inform why some individuals may have differential outcomes in response to the same interventions. Historically, interventions for autistic individuals were designed and implemented in research or clinical settings, followed by efficacy trials that eventually culminated in effectiveness studies in the community (8, 9). However, without stakeholders' involvement and input at the onset of intervention development and implementation effective and sustainable outcomes are attenuated [e.g., (10)]. This research to practice gap occurs in part because efficacy studies often do not consider the social contexts, perceived needs, values, or beliefs of key stakeholders such as families or individuals with disabilities, which impact treatment acceptability (11, 12). Thus, the field of implementation science calls for the involvement of stakeholders throughout the development process to yield high-quality interventions for autistic individuals that are acceptable and feasible in real-life contexts (8, 13). Implementation science, design-based implementation research, and community-based participatory research are crucial to understand the pertinent factors and mechanisms necessary for increasing feasibility and acceptability in clinical and community settings, thereby improving implementation fidelity and treatment outcomes.

In this Research Topic *Innovation Informs Best Practices in Autism Intervention Across the Lifespan*, articles addressed three unique intervention or service domains for individuals with ASD (behavior, secondary transition, and language). More importantly, these articles employed innovative methods (i.e., Linguistic Inquiry and Word Count analysis, collaborative autoethnography, and Expressive Language Sampling Narrative procedure) in investigating pertinent factors to advance these intervention or service domains to improve acceptance, feasibility, delivery, and fidelity.

The first study, *Applied Behavior Analysis as Treatment for Autism Spectrum Disorders: Topic Modeling and Linguistic Analysis of Reddit Posts* was conducted by Bellon-Harn et al. Despite positive empirical support of Applied Behavioral Analysis (ABA) in early intervention in autism [e.g., (14)] and for short-term cognitive and adaptive behavioral outcomes [e.g., (15)]; little is known about the perceptions of ABA in the public sphere. It is critical to understand factors that influence intervention effectiveness and acceptability. The authors investigated the perspectives of ABA from professionals, parents, and autistic individuals using the Linguistic Inquiry and Word Count (LIWC) tool. Results showed that perceptions were mostly geared toward personal experiences and opinions, and little was mentioned about clinical and research information. The authors link themes related to families and autistic individuals' opinions and experiences with ABA-based interventions to help practitioners support families during this decision process.

The second article entitled *Parent-researcher Perspectives on Role Intersectionality Related to Autism Research* by Hall et al. Family caregivers often provide lifelong care and support for individuals with ASD, thus, caregivers often become a central part of the life of autistic individuals (16, 17). Hall et al. capitalized on the participants' roles as both parents and researchers to serve as a resource for others, by understanding the benefits and barriers of having parents who are professionals and are involved as a researcher in conducting research in autism. Using collaborative autoethnography in-depth qualitative interview, results shed light on parent-researchers' unique perspective in ASD research, highlighting bias toward parents and having parents as assets in autism focused research.

The final article entitled *Using Telehealth-delivered Procedures to Collect a Parent-implemented Expressive Language Sampling Narrative Task in Monolingual and Bilingual Families with Autism Spectrum Disorder: A Pilot Study* by del Hoyo Soriano et al. examined the feasibility of teaching English-speaking and Spanish-speaking parents to administer the Expressive Language Sampling Narrative (ELS-N) procedures to their children at home through telehealth methods. This innovative method overcomes the limitations of norm-referenced standardized tests to assess autistic individuals with varying degrees of language abilities and performance across different domains (18, 19) by using pictorial methods of assessment that are age and developmentally appropriate (20). The study provides additional evidence to support the validity of the measure and has important implications for families as a collaborative partner in the monitoring of treatment progress.

This special topic addresses autism intervention in diverse topical areas, including applied behavior analysis, language, and parents as agents of research. While ABA has long been established as an evidenced-based practice, recent discussions have raised controversies due to misalignment between the values and philosophies of ABA and the perceived needs of the autism community, which may affect buy-in and sustainability. The use of parents as an implementer and researcher is an innovation to practice, as parents and family caregivers often hold unique knowledge that is valuable in contributing to the planning and care of the individual with autism. These articles serve as a springboard for future research to bridge the research-practice gap in clinical and community settings.

## Author contributions

All authors listed have made a substantial, direct, and intellectual contribution to the work and approved it for publication.

## Conflict of interest

The authors declare that the research was conducted in the absence of any commercial or financial relationships that could be construed as a potential conflict of interest.

## Publisher's note

All claims expressed in this article are solely those of the authors and do not necessarily represent those of their affiliated organizations, or those of the publisher, the editors and the reviewers. Any product that may be evaluated in this article, or claim that may be made by its manufacturer, is not guaranteed or endorsed by the publisher.
